# Targeting the diuretic hormone receptor to control the cotton leafworm, Spodoptera littoralis

**DOI:** 10.1093/jis/14.1.87

**Published:** 2014-07-08

**Authors:** Fabio Apone, Alessandra Ruggiero, Assunta Tortora, Annalisa Tito, Maria Rosaria Grimaldi, Stefania Arciello, Davide Andrenacci, Ilaria Di Lelio, Gabriella Colucci

**Affiliations:** 1 Arterra BioSci., via Brin 69, 80142 Napoli, Italy; 2 Current address: Center for Cardiovascular Genetics, The University of Texas Health Science Center, 6770 Bertner Street, Houston, TX 77030; 3 Institute of Genetics and Biophysics, CNR, via Castellino 111, 80131 Napoli, Italy; 4 Department of Entomology and Agricultural Zoology, University of Napoli, via Università 100, 80055 Portici (NA), Italy

**Keywords:** insect pest, G protein coupled receptors, RNA interference, molecular target

## Abstract

The cotton leafworm, *Spodoptera littoralis* Boisduval (Lepidoptera: Noctuidae), is one of the most devastating pests of crops worldwide. Several types of treatments have been used against this pest, but many of them failed because of the rapid development of genetic resistance in the different insect populations. G protein coupled receptors have vital functions in most organisms, including insects; thus, they are appealing targets for species-specific pest control strategies. Among the insect G protein coupled receptors, the diuretic hormone receptors have several key roles in development and metabolism, but their importance *in vivo* and their potential role as targets of novel pest control strategies are largely unexplored. With the goal of using DHR genes as targets to control *S. littoralis,* we cloned a corticotropin-releasing factor-like binding receptor in this species and expressed the corresponding dsRNA in tobacco plants to knock down the receptor activity *in vivo* through RNA interference. We also expressed the receptor in mammalian cells to study its signaling pathways. The results indicate that this diuretic hormone receptor gene has vital roles in *S. littoralis* and represents an excellent molecular target to protect agriculturally-important plants from this pest.

## Introduction

The cotton leafworm, *Spodoptera littoralis* Boisduval (Lepidoptera: Noctuidae), feeds on a wide range of important crops and is one of the most destructive pests in the tropical and subtropical areas of the world ( Hill 1987 ). To control the attacks of this insect, several types of insecticides have been used, including synthetic pyrethroids, organophosphates, and nonsteroidal compounds ( Casida and Quistad 1998 ). The extensive use of these insecticides has caused resistant insect strains to emerge ( Davies et al. 2007 , Mosallanejad and Smagghe 2009 ) and serious toxicological problems to humans and the environment ( Costa et al. 2008 , Relyea 2009 ).

For some lepidopteran species, such as *Manduca sexta* (L.) and *Helicoverpa zea* (Boddie), the most successful strategy of control has been to use transgenic plants expressing Bt toxins ( Kota et al. 1999 ), but *Spodoptera* spp. have shown very low or negligible sensitivity toward these proteins ( Sivasupramaniam et al. 2008 ). Recent studies have reported the presence of different types of resistance to Bt toxins among *Spodoptera* populations exposed to transgenic plants in the field ( Tabashnik et al. 2008 ). A more effective, environmentally-sustainable control of *S. littoralis* could be the use of alternative strategies that target specific molecules of this insect and do not produce any toxicity to natural ecosystems or humans.

G protein coupled receptors (GPCRs) constitute a large family of proteins in all animals and have essential functions in most body tissues in insects. They are receptors with 7-transmembrane domains and use heterotrimer-ic G proteins to transduce their signal into the cells by activating enzymatic effectors localized at the plasma membrane and/or in the cytoplasm ( Marinissen and Gutkind 2001 , Woehler and Ponimaskin 2009 ). The range of ligands, including hormones, neurotransmit-ters, ions, aminoacids, and light that signals through GPCRs, underscores the importance of this class of receptors in regulating cellular activities and metabolism.

Human GPCRs are a primary focus of bio-medical research and pharmaceutical drug discovery programs ( Thomsen et al. 2005 , Allen and Roth 2011 ). In insects, where more than 250 GPCR genes have been identified, GPCRs may be attractive targets for novel insecticides and pest control strategies. Most of the attention has been given to the Octopamine receptors, which are used in screening procedures to identify new insecticides ( Ohta et al. 2012 ).

Among the GPCRs, the neuropeptide receptors are attractive targets for insects because of their fundamental roles in regulating vital physiological processes ( Coast et al. 2001 ). In particular, the receptors that bind the cortico-tropin-releasing factor-related diuretic hormone receptor (DHR) regulate different key functions in the insect development and metabolism, including water and ion balance, excretion, and feeding behaviour (see Gade and Goldsworthy 2003 , Schooley et al. 2012 for review). By the analysis of *Bombyx mori* (L.) genome, eight putative genes belonging to the secretin-like receptor family (class B) were identified in Lepidoptera ( Fan et al. 2010 ). Among them, four were reported to be similar to vertebrate calcitonin receptors and to *Drosophila* CG17415 and CG13758 ( Johnson et al. 2005 , Mertens et al. 2005 ); another, BmDHR (Ha et al. 2000), was previously characterized as the homologue of two *Drosophila* CRF-like binding DHR, CG8422 and CG12370 ( Hector et al. 2009 ).

To explore the use of a CRF-like binding DHR as target for insect control, we cloned the homologue of BmDHR gene in *S. littoralis* and studied its potential role in vivo by using RNA interference (RNAi) technology. The most successful results were obtained by feeding *S. littoralis* caterpillars on transgenic tobacco *(Nicotiana tabacum* L. (Solanales: Solanaceae)) plants expressing double strand RNA (dsRNA), corresponding to the DHR gene. The results of this study underscore the vital importance of CRF-like binding DHR in *Spodoptera* spp. and provide novel ways to protect agriculturally important plants from the attacks of this lepidopteran.

## Materials and Methods

### Growth of *S. littoralis*

*S. littoralis* larvae were maintained at 23°C and 70% RH with a 16/8 h L/D photoperiod. They were fed an artificial diet composed of 41.4 g/L wheat germ, 59.2 g/L brewer’s yeast, 165 g/L corn meal, 5.9 g/L ascorbic acid, 1.53 g/L benzoic acid, 1.8 g/L methyl 4-hydroxybenzoate, and 29.6 g/L agar. Sixth instars were transferred into plastic boxes containing vermiculite to let them reach the pupa stage. The adults were mated to get new generations of larvae.

### Cloning of a *S. littoralis* CRF-like binding DHR and RT-PCR experiments

To isolate the full ORF (open reading frame) encoding a CRF-like binding DHR from *S. littoralis,* protein sequences of *Bombyx mori, Acheta domesticus* (L.) *, Drosophila melanogaster* Meigen, and *Tribolium castaneum* (Herbst) CRF-related DH receptors were retrieved from the GenBank database or literature and aligned using the program "Multiple sequence alignment with hierarchical clustering” ( Corpet 1988 ). The alignment highlighted all the protein regions containing identical amino acids. The regions containing the highest number of identical aminoacids in a row and the lowest degree of degeneration of the corresponding nucleotide sequences were chosen to design the degenerate oligonucleotides for the cloning.

Total RNA was extracted from fifth instars of *S. littoralis* by using the SV Total RNA Isolation kit (Promega). cDNA was synthesized by using the RevertAid Reverse Transcriptase (Fermentas Int.) at the following reaction conditions: 70°C for 5 min, 37 °C for 10 min, 42°C for 1 hr, and 72°C for 10 min. The cDNA was used in a standard PCR reaction with the following degenerate primers:

DHR-Fw, 5'-TTYYTNTAYTTYAARGANY TNMGNTGY-3'

DHR-Rv, 5'-ARYTTNGTNATNARNACCC ACATNAT-3'

corresponding to the aminoacid sequences FLYFKDLRC and IMWVLITKL) at concentration of 2.5 mM. The PCR program included a denaturation step of 2 min at 98°C, followed by 35 cycles of 98°C for 10 sec, 55°C for 30 sec, 72°C for 30 sec, and a final extension step of 10 min at 72°C. The amplified 500 bp fragment was cloned into the TA-TOPO vector (Invitrogen) and sequenced. To get the full-length ORF, a 5'/3' Race PCR was performed by using gene specific primers and anchor primers supplied in the 5'/3' RACE-KIT (Boeringer Ingelheim).

For the 3'Race, the following oligos were used:

5'-AACCTCATGTCGACGTATATTCTGTC T-3'

5'-ATGCTTGTAGAAGGTTTGTACCTGTA C-3'

5'-TGGGTTATATGCAGGTGCTTCGTCAA C-3'.

For the 5’ Race, the following oligos were used:

5'-CATACATATGACCAGAATCGTACAC GA-3'

5'-GGCGAGGTAGATGAGGCTGGTGACG TC-3'.

The 3' and 5' Race reactions gave fragments of 800 and 500 bp, respectively, with 40 cycles of amplification consisting of 10 sec at 98°C, 30 sec at 46°C, 60 sec at 72°C, followed by 10 min of final extension at 72°C. The full-length ORF was obtained by amplifying with the following specific primers:

5'-ATGGCGGAGAAGTGCCTGGCG-3'

5'-TCATACCGTGAGTCGTATGCT-3'.

For the analysis of the *S. littoralis* DHR transcript level in the different developmental stages and after microinjection with dsRNA, total RNA was extracted from three to 10 larvae, and cDNA was synthesized as described above.

The sequences of the primers used for quantitative DHR mRNA analysis were:

DHRqFw: 5'-ATGGCGGAGAAGTGCCTGG CG3'

DHRqRev: 5'-ACCACGAGCATGTACAGG TAC-3'

These gave a fragment of 560 bp. As a control, the *S. littoralis* β-actin transcript was amplified by PCR using the same cDNA template and the following actin specific primers:

SlActinFw: 5′-GCGTCGCCCCTGAGGAAC AC-3′

SlActinRv: 5′-CGACGTACATGGCGGGGG AG-3′.

The typical scheme used for the amplifications was: 94°C for 2 min, followed by 30 cycles of 30 sec at 94°C, 30 sec at 55°C, 30‒60 sec at 72°C. All the PCR reactions were performed by using the enzyme Taq DNA polymerase (Euroclone) in the master cycler Ep-gradients (Eppendorf). The PCR products were analyzed on a 1% agarose gel, stained with 0.5 μg/mL ethidium bromide, and displayed with the Geliance 200 Imaging system (Perkin Elmer).

### Plant growth and transformation

*Nicotiana tabacum* plants, cv. Samsun NN, were grown in a growth chamber at 24°C, light intensity of 250 μEinsten m-2s-1, 16/8 h L/D, and 70% RH. To produce the transgenic lines, the full *S. littoralis* DHR coding sequence was subcloned into the plant expression vector pH7GWIWG2(I) using the gateway technology. To produce the transgenic lines, the full coding sequence was cloned into the plant expression vector pH7GWIWG2(I), downstream of the promoter 35 S, by using the gateway technology (Invitrogen), as described by Karimi et al *.* (2005) . The DHR coding sequence was first cloned into the gateway entry-vector pENTR 2B by using restriction endonucleases and ligase. Then, the gene was subcloned into the destination binary vector pH7GWIWG2(I) by sing the enzyme clonase, which performed homologous recombination twice by recognizing specific sites present in the entry and the destination vectors. The resulting construct was a vector containing two DHR sequences, one in the 5′‒3′ and the other in the 3′‒5′ orientation downstream of the promoter, separated by an intronic sequence. Once transcribed in the plant, the resulting mRNA would fold back on itself due to the pairing of the sense and antisense sequences and form a double-stranded RNA molecule with a hairpin structure.

The vector pH7GWIWG2(I), carrying the DHR sequences, was introduced into *Agrobacterium tumefaciens* strain C58 ( Binns and Thomashow 1988 ) and used to transform the plants, according to the protocol described by Corrado et al. (2007) . The transformed plants were analyzed by RT-PCR to verify the presence of the specific transgene and, whether positive, propagated to fresh medium and grown in soil.

### RT-PCR analysis of *Nicotiana tabacum* plants

To verify the presence of the transgene in tobacco shoots or plants, total RNA was extracted from 50 mg of fresh tissue using Genelute Mammalian Total RNA kit (Sigma-Aldrich). To avoid the presence of any residual DNA, each RNA sample was treated with five units of Qualified RNAse-free DNAse (Promega) for 30 min at 37°C. The cDNA was synthesized using 2 µg of total RNA with RevertAid M-MuLV Reverse Transcriptase (Fermentas Int.). All PCR reactions were carried out using the ribosomal RNA QuantumRNA 18S Internal Standards (Ambi-on) as internal control. The primers and the conditions used for the RT-PCR reactions were identical to those described for the analysis of DHR expression in the insect.

### Microinjection of *S. littoralis* larvae with dsRNA

The gene fragment of 560 bp (obtained by amplifying with the oligos DHRqFw and DHRqRev) was used to synthesize the dsRNA in vitro by the Megascript RNAi Kit (Ambi-on). Fifth instars (day 2) were immobilized by carbon dioxide and injected with 3 µg of dsRNA, which was delivered into the haemolymph by the ventral part of the insect abdomen by using a Hamilton syringe (needle 4, gauge 32). As control, equivalent numbers of larvae were injected with 500 bp dsRNA fragments synthesized from a DNA template provided in the kit. After 48 hr, total RNA was extracted and processed as described above. The results of the phenotypic analysis and mortality rate were analyzed by the *t-* test for paired samples for statistical significance.

### Feeding bioassays of *S. littoralis* larvae on transgenic tobacco plants

**Figure 5.**
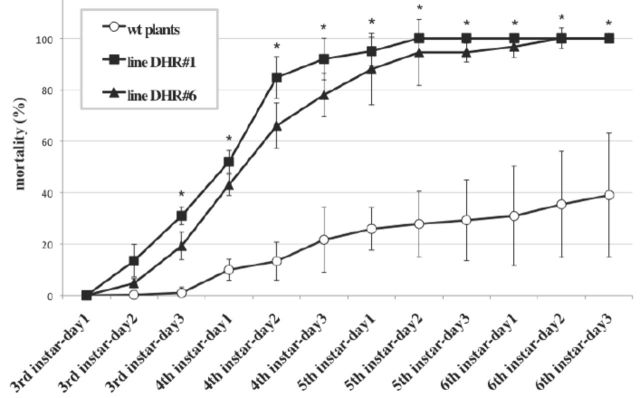
Daily percentage of mortality of *Spodoptera littoralis* larvae. Starting with third instars, the larvae were fed on transgenic Spoli-DHR. dsRNA plants, belonging to line #1 (full squares) and line #6 (full triangles), and on wild type plants (empty circles). The mortality rate was measured every day until the pupa stage. The values are the averages of four experiments, and the error bars indicate standard deviations (* *P* < 0.01). High quality figures are available online.

Two hundred first instars were fed an artificial diet from egg hatching to the end of the second stadium. At the beginning of the third stadium, 60 larvae for each condition were collected and placed separately in a Petri dish containing 0.5 g of fresh leaf laid on 2% agar to keep it moist. Every day, freshly-cut leaves were provided to the larvae, and the percentage of mortality was measured. The percentage values reported in Figure 5 were the averages of measurements obtained from four independent experiments. The statistical significance of the measurements was analyzed by the *t-* test for paired samples at the *P* < 0.01 level.

### Real-time PCR

500 ng of extracted RNA was treated with Qualified RNAse-free DNAse (Promega) for 30 min at 37°C and reverse-transcribed by using the QuantiTect Reverse Transcription kit (Quiagen). To minimize variations during the cDNA synthesis step, all RNA samples were reverse-transcribed simultaneously. The sequences of the primers used for the amplification of the *Spodoptera* DHR genefragments were Fw:5'-GACGGCGTGTGGCACAACTAC-3' and Rv: 5'-CGGCGAGGCTGAGCGAGTAG-3'. The size of the fragment obtained was 117 bp. Real-time PCR reactions were performed in triplicate in 20 µL reaction volumes, and the general protocol described in Conte et al. (2010) was followed. For each transcript, the qRT-PCR values obtained, recorded as threshold cycle numbers, were analyzed by means of the ABI Prism 7900HT Fast Sequence Detection System software (Applied Biosystem), normalized against an internal control (β-actin), and then expressed as percentage values to control.

### Cell transfection, cAMP, and calcium measurements

Chinese hamster ovary (CHO) cells were transfected with lipofectamine (Invitrogen) using 10 µg DNA per 4 × 10 ^6^ cells. Stable lines expressing *Spodoptera* DHR were generated through selection of resistance to the antibiotic G418. Cells were maintained in a humidified incubator under 5% CO _2_ atmosphere at 37°C and split 1:5 every three days. The growth medium was Dulbecco’s modified eagle medium (DMEM) supplemented with 10% fetal bovine serum (FBS) and antibiotics.

To measure cAMP, the cells, stably expressing the receptor, were washed with PBS 1X (136 mM NaCl, 2.7 mM KCl, 12 mM NaH _2_ PO _4_ , and 1.76 mM KH _2_ PO _4_ , pH 7.4), detached from the flasks with a non-enzymatic dissociation solution (Sigma-Aldrich), and resuspended at the concentration of 10 ^6^ /mL in stimulation buffer (BSA 0.1%, IBMX 0.5% in PBS 1X), containing Alexa Fluor 647-labeled anti-cAMP antibody, according to the protocol described in the Lance kit (Perkin Elmer). 12,000 cells were distributed in aliquots in 384-well plates and treated with only buffer, different concentrations of *Manduca sexta* (L.) diuretic hormone (DH _41_ ) (Phoenix Pharmaceuticals) and 5 µM forskolin (Sigma-Aldrich). In parallel, a standard curve for cAMP was prepared by diluting known concentrations of cAMP in stimulation buffer, in the presence of anti-cAMP antibody. The cells were incubated for 1 hr at room temperature and then lysed in 12 mL of detection mix (provided in the kit), containing Europium-W8044-labeled strep-tavidin and biotin-labeled cAMP. The amount of cAMP produced by the cells was measured by exciting at 320 nm and recording at 615 and 665 nm by the instrument EnVision (Perkin Elmer).

**Figure 7.**
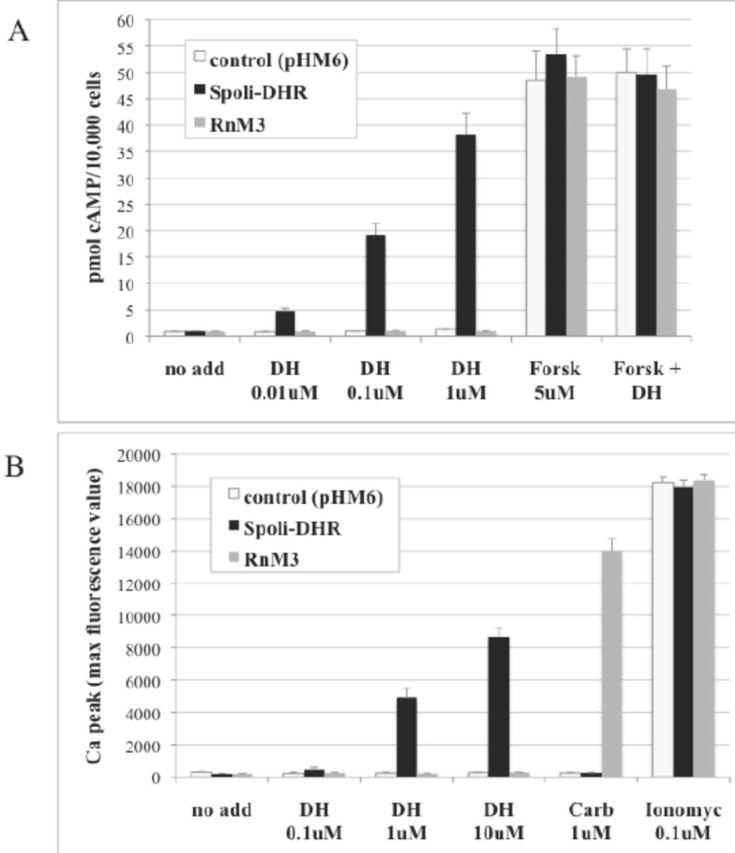
Measurement of cAMP (A) and calcium (B) content in CHO cells. Cell lines stably expressing Spoli-DHR and *Rattus norvegicus* Muscarinic Receptor 3 (RnM3) were treated with the indicated agonists and the levels of the two second messengers were measured. Either for cAMP or calcium assay, one representative experiment was reported and error bars represented standard deviations of five independent measurements. DH, *Manduca sexta* DH41; Forsk, forskolin; Carb, carbachol; Ionomyc, ionomycin. High quality figures are available online.

For calcium measurements, cells were loaded with 5 µM of the calcium-sensitive fluorescent dye FLUO3-AM (Molecular Probes), dissolved in a Hank’s balanced salt solution (HBSS) containing 20% Hepes buffer, 2.5 µM probenecid, and 0.02 % pluronic acid (Sigma-Aldrich). After 45 min incubation at 37°C, the cells were washed three times with HBSS/Hepes/probenecid and dispensed into 96-well plates containing the agonists: different concentrations of *M. sexta* diuretic hormone (DH _41_ ) (Phoenix Pharmaceuticals), 1 µM carbacol or 0.1 µM ionomycin (Sigma-Aldrich). Every second for 2 min after dispensing the cells, the fluorescence at 535 nm (excitation at 495 nm) was recorded by the microplate fluorescence reader Envision (Perkin Elmer), and the peak values of each set of measurements were reported in Figure 7B .

## Results and Discussion

### Cloning and characterization of a CRF-like binding DHR gene in *S. littoralis*

**Figure 1.**
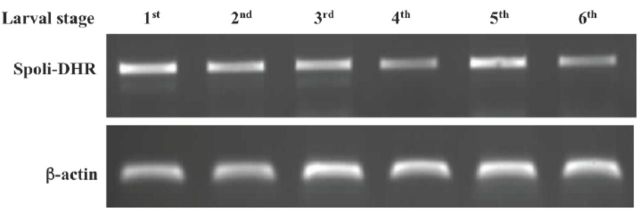
Spoli-DHR gene expression in the different larval stages of *Spodoptera littoralis.* For each stage, three to five larvae were frozen in liquid nitrogen and RNA was extracted. The synthesized cDNA was used as template in PCR reactions by using specific oligonucleotides. The band of around 500 bp (upper panel) corresponds to the DHR gene. The gene of the pactin (lower panel) was used as a comparative standard to quantify the expression of the DHR gene. High quality figures are available online.

**Figure 2.**
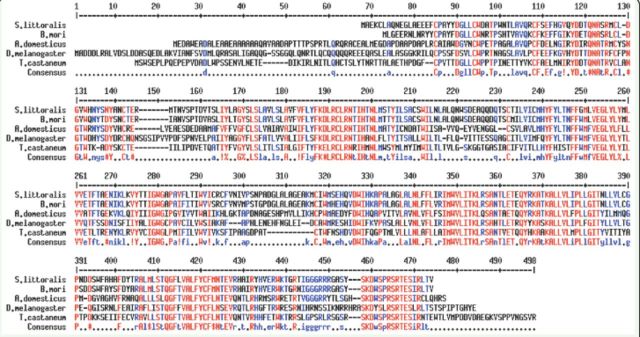
Alignment of the DHR protein sequences belonging to the species: *Spodoptera littoralis* ACJ06650.1; *Bombyx mori* (Ha et al. 1999), *Acheta domesticus* AAC47000.1; *Drosophila melanogastar* AAY55823.1; *Tribolium castaneum* NP001 1 67548.1. To make the alignment, the program "Multiple sequence alignment with hierarchical clustering” ( Corpet 1988 ) was used. High quality figures are available online.

Based on the alignment of the CRF-related DH receptor sequences of *Bombyx mori, Acheta domesticus, Drosophila melanogaster* , and *Tribolium castaneum* present in the Gene bank (except the one of *B. mori* , which was retrieved from Ha et al. 2000), degenerate primers in the conserved regions FLYFKDLRC and IMWVLITKL were designed and used in PCR reactions on *S. littoralis* cDNA, extracted from larvae at different stages of development. After cloning a first gene fragment of 500 bp, the expression of the DHR gene in all the larval stages was analyzed by using specific primers in PCR reactions and compared with the constitutive expression of β-actin. The results, shown in Figure 1 , indicate that the DHR transcript level, measured in whole bodies, was constant through the whole larval cycle of *S. littoralis* . On this basis, new cDNA from fifth instars was synthesized and used to obtain the whole coding sequence (1190 bp) by 5'and 3'Race PCR. The gene sequence of *S. littoralis* DHR was deposited in the GenBank with the accession number FJ374690 and was named Spoli-DHR on the basis of the recently proposed consensus nomenclature for insect genes ( Coast and Schooley 2011 ). The analysis of the protein sequence by the algorithm TMHMM Server v. 2.0 ( http://www.cbs.dtu.dk/services/TMHMM ) revealed the presence of the seven transmembrane domains and a classical GPCR topology with an extracellular N-terminus and a cyto-solic C-terminus. The comparison of Spoli-DHR protein sequence with those of other insects showed a high percentage of similarity among the lepidopteran species (∽90%), and 52-53% with other insect orders ( Figure 2 ).

### RNA interference experiments on *S. littoralis* larvae

RNAi is one of the most successful techniques used to evaluate the roles of genes. Recent data showed that it can be a valuable method to knock down genes even in lepidopteran species ( Terenius et al. 2011 ). For species such as *Bombyx mori* and *Manduca sexta* , the efficiency of silencing for Hox genes and V-type ATPase reached very good levels either by injecting dsRNA into the embryos (Masumoto et al. 2009) or by feeding the larvae with dsRNA ( Whyard et al. 2009 ). For *Spodoptera* spp., RNAi by feeding also gave positive results for the genes of allatostatins ( Griebler et al. 2008 ), and for those involved in chitin synthesis ( Tian et al. 2009 ) and sugar metabolism ( Tang et al. 2010 ).

To investigate the role of Spoli-DHR in *S. littoralis* , we performed a series of experiments aimed at reducing the expression of this receptor *in vivo* by using RNAi. The dsRNA corresponding to the Spoli-DHR gene was introduced in the insect larvae by two delivery methods, microinjection and feeding.

**Figure 3.**
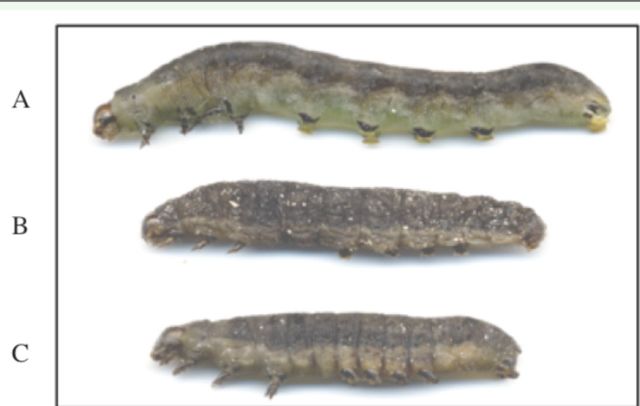
Photograph of *Spodoptera littoralis* interfered larvae. Fifth instars were injected with dsRNA and observed after 48 h. A, larva injected with the dsRNA control template; Band C, larvae injected with 3 mg of Spoli-DHR dsRNA. High quality figures are available online.

In the first set of experiments, larvae (from 30 to 60 for each condition) were injected with 3 µg of *in vitro* synthesized Spoli-DHR dsRNA (560 bp fragment) and observed for potential morphological changes and mortality rate. Only in six of 15 experiments did the larvae injected with Spoli-DHR dsRNA show significant differences, in terms of phenotype and mortality rate, compared with those injected with the control template. In these experiments, we observed an average percentage of mortality of 17.9% ± 5.4 in the larvae injected with the Spoli-DHR-dsRNA before reaching the pupal stage, compared with 5.8 % ± 4.6 in those injected with the control template. A statistical analysis of the results gave a *P* value of < 0.05. Moreover, 10.2% ± 3.4% of the Spoli-DHR dsRNA injected larvae showed an evident phenotype that was not observed in the controls. They became lethargic, stopped eating, and before dying, they appeared dryer and thinner than the control larvae ( Figure 3 ).

These observations agreed with the findings of Keeley et al. (1992) , who showed that water excretion and food consumption were dramatically altered in *Heliothis virescens* larvae by exogenously administered CRF-like diuretic peptide. In the same article, the authors showed that non-physiological amounts of diuretic peptide could regulate the water balance and the feeding behavior of the larvae and create significant changes in the body weight.

RT-PCR analysis conducted on five larvae injected with the dsRNA showed that the amount of mRNA corresponding to Spoli-DHR gene was reduced after 48 hr by 13.8% ± 9.1% compared with control larvae (data not shown). Unfortunately, these results were not always reproducible, meaning that the phenotypic effects of lethargy, feeding, and dryness were not always evident. As reported above, only 40% of the injections gave consistent results, which was attributed to the type of technique used to deliver the dsRNA.

To overcome these difficulties, we decided to produce dsRNA-expressing tobacco plants to feed the larvae and evaluate their effect on the development and viability of the larvae. Because of its high versatility to genetic manipulation, tobacco is a good system to study RNAi by feeding and has been used successfully to knock down gene expression in lepidopteran species, including *Spodoptera* ( De Leo et al. 1998 , Zhu et al. 2012 ).

**Figure 4.**
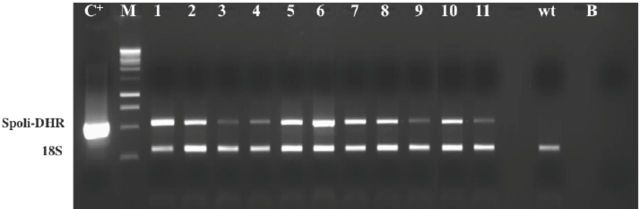
RT-PCR of transgenic tobacco plants expressing Spoli-DHR dsRNA. Spoli-DHR 500 bp fragment corresponding to *Spodoptera littoralis* DHR gene; 18S, internal standard. Lanes: C ^+^ , pENTR-2B+DHR gene (positive control of the PCR); M, 1kb DNA ladder; 1-11, samples of the transformed plants; wt, untransformed tobacco plants; B, blank (negative control of the PCR). High quality figures are available online.

The entire Spoli-DHR coding sequence was subcloned into a plant expression vector that, once in the plant cells, produced stable molecules of dsRNA corresponding to the gene of interest. After transformation by *Agrobacterium tumefaciens* , 20 tobacco transformants (T0) were selected and checked for the presence of the transgene by isolating the RNA and performing RT-PCR with specific primers. As shown in Figure 4 , 11 plants had a detectable level of the specific transgene, in the form of expressed dsRNA. In particular, the transgenic lines #1 and #6 (i.e., those with the highest expression level) were propagated and used for all of the following tests.

In the feeding bioassays, 60 third instars were transferred into plastic boxes containing leaf disks, excised from either wild type or trans-genic Spoli-DHR plants. Mortality was measured daily for 12 days until the pupa formation. The experiment was repeated four times, and the results were always consistent.

The values measured ( Figure 5 ) indicate that the mortality rate of the larvae fed the trans-genic leaves, derived from lines #1 and #6, was significantly higher than that calculated for the larvae fed on wild type plants. Seven days after being fed the transgenic plants (corresponding to day 2 of the fifth instars), the mortality of the larval populations reached almost 100%, compared with 25% for the controls. Similar phenotypes to those found in the microinjection experiments were observed. Larvae fed transgenic leaves were smaller and thinner; they consumed much less and ate slower than the control larvae ( Figure 3 ).

**Figure 6.**
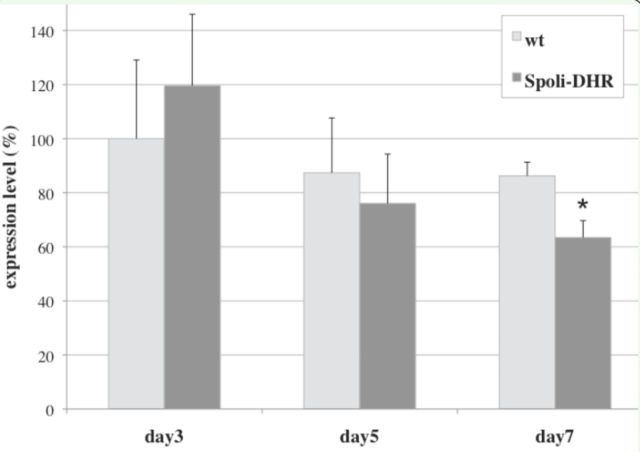
Spoli-DHR gene expression level of *Spodoptera littoralis* larvae analyzed by real-time PCR. Larvae fed on wild type plants (light columns) and transgenic plants (dark columns) were collected at days 3, 5, and *7,* and total RNA was extracted for the quantitative analysis. The values are averages of three experiments, and error bars indicate standard deviations (* *P* < 0.01). High quality figures are available online.

To verify whether the effect of mortality observed in the bioassays was associated with a reduction of the specific mRNA caused by the interference, we measured the transcript level of Spoli-DHR gene in the interfered larvae by RT-PCR and compared it with that found in the control larvae fed on wild type plants. For this analysis, five larvae were assayed at days 3, 5, and 7 from the beginning of the feeding experiment, and total RNA was extracted. We observed a 27% reduction of Spoli-DHR mRNA level in the larvae fed the transgenic leaves compared with control larvae ( Figure 6 ). This reduction was only detectable after seven days of feeding, although the effect on the larval vitality became visible a few days after the start of the feeding with the transgenic plants. This can be explained by the fact that in the first week of treatment, the reduction of Spoli-DHR transcript level might be small or not detectable in the whole larval bodies, even though it is sufficient to determine a phenotypic effect on the larval vitality. After prolonged treatments of the larvae with the dsRNA, however, the level of the Spoli-DHR transcript may decrease further in specific target tissues, thus becoming detectable even in total bodies. Unlike in other organisms, such as *C. elegans* ( Tijsterman et al. 2004 ), a systemic RNAi has been quite difficult to achieve in Lepidoptera, and wide variations in RNAi efficiency related to tissues were reported (see Terenius et al. 2011 for review). In any case, our results show that the reduction of Spoli-DHR transcript level caused by RNAi, although modest, was always significant and sufficient to cause a high level of mortality in the larval populations of *S. littoralis* .

### Expression of Spoli-DHR in mammalian cells and bioassays

Having validated Spoli-DHR as a target in *Spodoptera* , this receptor was expressed in mammalian cells in order to study its signaling trasnduction pathway. The whole Spoli-DHR coding sequence was cloned into pHM6 expression vector downstream of the sequence of the Hemo-Agglutinin (HA) epitope and transfected in CHO (Chinese hamster ovary) cells. The receptor expression level was determined by ELISA assay, which was carried out on fixed cells by using a commercial anti-HA antibody. CHO stable lines expressing Spoli-DHR were produced to have a constant and homogeneous expression of the receptor in the cells and to standardize the screening conditions. The study of the signal transduction pathways activated by the receptor showed that the second messengers’ cAMP and calcium were involved in the DHR signaling ( Figure 7 ). To confirm the specificity of the measurements, in each experiment the cells also were treated with the compounds forskolin and ionomycin, which stimulated the production of cAMP and calcium, respectively. For comparison, the treatments were conducted on a cell line expressing another GPCR, the *Rattus norvegicus* Muscarinic receptor 3 (RnM3), which is activated by the agonist carbachol and involved in calcium signaling ( Cheng et al. 2002 ). The study demonstrated that Spoli-DHR could be functionally expressed in mammalian cells and activated by the agonist CRF-related DH, which produced increases of cAMP and calcium levels. Although cAMP could be significantly induced by concentrations of DH <1 µM, the stimulation of calcium levels needed higher DH concentrations, which agrees with previous studies on the *Drosophila* DH receptor CG8422 ( Johnson et al. 2004 ).

## Conclusions

In this study we demonstrated that feeding *S. littoralis* caterpillars on the plants, expressing the dsRNA corresponding to a CRF-like binding DHR, decreased the transcription level of the target gene and caused an increase of the mortality rate in the insect populations. RNAi technology by feeding has been used in several studies to knock down genes in *Spodoptera* spp. ( Terenius et al. 2011 ), but there are few examples of using transgenic dsRNA-expressing plants ( Zhu et al. 2012 ). In this article, we believe for the first time, we describe the use of a dsRNA in plants, which is directed to reduce the expression of a GPCR gene *in vivo* . Several studies have underlined the involvement of GPCR genes in many vital processes in Lepidoptera, but none have reported *in vivo* analyses to validate their functions.

Our results suggest that the regulation of DHR expression in *Spodoptera* spp. is essential for the insect’s survival, and this receptor, which is constantly expressed during the larval development, can be an easy target of RNAi and is a good candidate gene for insect control.

Although the most widely used method to deliver dsRNA in Lepidoptera is still microinjection ( Quan et al. 2002 ), in our experiments with *S. littoralis* , this method did not give reproducible results. This agrees with the high range of variability observed in RNAi experiments with different lepidopterans (unpublished data, discussed in Terenius et al. 2011 ) and the unsuccessful attempts to knock down genes involved in hormone signaling in *S. littoralis* (Iga and Smagghe 2010). *Spodoptera* larval stages are often refractory to RNAi by microinjection and may have different susceptibility depending on the type and the expression level of the gene to knock down. We found, however, that the effectiveness of RNAi technology using dsRNA-expressing plants is much more robust and reliable and gives consistent results.

In conclusion, our results suggest that Spoli-DHR has vital importance for *S. littoralis* and thus can be exploited as target of new pest control strategies. Two potential applications emerged from this study: the use of transgenic plants expressing dsRNA of Spoli-DHR gene and the development of a high throughput screening (HTS) platform to search for potential modulators of Spoli-DHR activity. Indeed, the calcium and the cAMP assays can be easily converted into HTS procedures to search for modulators of the Spoli-DHR activity. Because they are fast and reliable, these types of assays allow the handling and the identification of potential “hits” and may represent novel and selective agrochemicals to use in the control of *S. littoralis* .

## Abbreviations

DHR: diuretic hormone receptors
GPCRs: G protein coupled receptors
CRF: corticotropin-releasing factor
